# A host dTMP-bound structure of T4 phage dCMP hydroxymethylase mutant using an X-ray free electron laser

**DOI:** 10.1038/s41598-019-52825-y

**Published:** 2019-11-08

**Authors:** Si Hoon Park, Jaehyun Park, Sang Jae Lee, Woo Seok Yang, Sehan Park, Kyungdo Kim, Zee-Yong Park, Hyun Kyu Song

**Affiliations:** 1 Department of Life Sciences, Korea University, 145 Anam-ro, Seongbuk-gu, Seoul 02841 South Korea; 2 PAL-XFEL, Pohang Accelerator Laboratory, POSTECH, Pohang, Gyeongbuk 37673 South Korea; 3 School of Life Sciences, Gwangju Institute of Science and Technology, Gwangju, 61005 South Korea

**Keywords:** X-ray crystallography, Structural biology

## Abstract

The hydroxymethylation of cytosine bases plays a vital role in the phage DNA protection system inside the host *Escherichia coli*. This modification is known to be catalyzed by the dCMP hydroxymethylase from bacteriophage T4 (T4dCH); structural information on the complexes with the substrate, dCMP and the co-factor, tetrahydrofolate is currently available. However, the detailed mechanism has not been understood clearly owing to a lack of structure in the complex with a reaction intermediate. We have applied the X-ray free electron laser (XFEL) technique to determine a high-resolution structure of a T4dCH D179N active site mutant. The XFEL structure was determined at room temperature and exhibited several unique features in comparison with previously determined structures. Unexpectedly, we observed a bulky electron density at the active site of the mutant that originated from the physiological host (i.e., *E. coli*). Mass-spectrometric analysis and a cautious interpretation of an electron density map indicated that it was a dTMP molecule. The bound dTMP mimicked the methylene intermediate from dCMP to 5′-hydroxymethy-dCMP, and a critical water molecule for the final hydroxylation was convincingly identified. Therefore, this study provides information that contributes to the understanding of hydroxymethylation.

## Introduction

The X-ray free electron laser (XFEL) is a new fourth-generation light source that produces considerably bright X-ray pulses^1–4^. In contrast to X-rays generated by synchrotron sources, XFELs use femtosecond pulse with a brightness that is a billion times higher. Applications well suited to XFEL include data collection with submicron crystals at room temperature (RT), avoidance of radiation damage, and time-resolved crystallography^1,4–12^.

After the XFEL structures of photosystem I (PDB ID: 3PCQ)^13^ and the photosynthetic reaction center (PDB ID: 4AC5)^14^ were solved with the Linac Coherent Light Source (LCLS), many structures including G protein-coupled receptors for drug design and photoreactive proteins for molecular motion were determined using XFEL data collected at XFEL facilities^8,15–21^. Interestingly, the *in cellulo* structure of cathepsin B with a natively inhibited propeptide complex (PDB ID: 3MOR) has been reported^22^. Although protein structures using XFELs have been reported consistently, they are an extremely small portion of the overall deposited PDB (0.09%; 143 structures out of 155,618). In addition, the studied proteins are largely limited to some classes of proteins such as photoactivating proteins for time-resolved (TR) experiments, membrane proteins that are stable in the lipidic cubic phase (LCP), and robust model proteins^23,24^. The intermediate structures of the adenine riboswitch (PDB IDs: 5SWD, 5SWE, and 5E54) and β-lactamase with ceftriaxone (PDB IDs: 6B5Y, 6B5X, and 6B6A) have been challenges faced by time-resolved serial X-ray crystallography (TR-SFX)^25,26^. In contrast to the laser-triggered “pump-probe” technique, the TR-SFX at room temperature (RT) based on “mix-and-inject” is more generally applicable for understanding the enzyme mechanism^6–8^.

Herein, we described the XFEL structure of dCMP hydroxymethylase (dCH) from bacteriophage T4 (T4dCH) at 1.9-Å resolution using the Pohang Accelerator Laboratory (PAL) XFEL. The dCH is the first identified viral enzyme from T-even phage, and it gives viral phage the opportunity to escape the restriction-modification system of host bacteria by the hydroxymethylation of a cytosine base^27–30^. It belongs to the thymidylate synthase (TS) superfamily and catalyzes dCMP to hydroxymethyl-dCMP (hmdCMP) using 5,10-methylenetetrahydrofolate (5,10-mTHF) and water as a methyl and hydroxyl donor, respectively^31,32^. Previously, we reported the apo-form and binary and ternary complexes structures of T4dCH, and we suggested the unique mechanism of hydroxymethylation^33,34^. However, questions about the cytosine modification catalysis remain to be answered because the methylene intermediate structure has not been elucidated yet.

It is also unknown whether hydroxymethylation is associated with water-mediated hydrogen bond networks in the active site. In this study, we solved the structure of T4dCH D179N mutant by introducing a TS-like character^35,36^ using the XFEL technique. Interestingly, the substrate binding site of the mutant was occupied by an unidentified ligand that comes from the natural host (i.e., *E. coli*) for this bacteriophage enzyme. Through a cautious interpretation of the electron-density map and the mass spectrometry results, the unknown ligand was confirmed as dTMP. This dTMP complex structure can mimic the methylene intermediate state. Thus, it can be used to propose the detailed dCMP hydroxymethylation mechanism. From our XFEL-based native dTMP complexed T4dCH structure at RT, we can better understand the hydroxymethylation mechanism of nucleoside monophosphates.

## Results and Discussion

### Microcrystals of T4dCH D179N mutant

**Figure 1:**
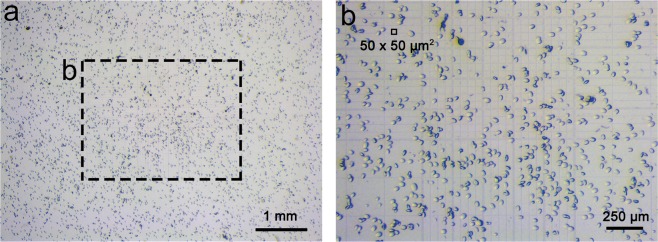
Microcrystallization of T4dCH D179N mutant. (**a**) Microcrystals observed in hemocytometer by light microscope. (**b**) Magnified images of microcrystals on Neubauer-improved chamber in hemocytometer. Size of small grid is 50 × 50 *μ*m^2^ as drawn in outlined box. See Supplementary Fig. 1 for flow cytometry results using these microcrystals.

Crystals of T4dCH D179N mutant nucleate faster than those of wild-type (WT) protein. Therefore, we attempted to focus on the crystallization of D179N microcrystals. Initially, we obtained D179N microcrystals by the vapor-diffusion method that was applied to solve the ternary complex structure^34^. However, there were several problems involved in the conventional crystallization method for obtaining the microcrystals. First, it was not sufficient to obtain a large amount of microcrystals owing to the spatial limitation of the drop size (up to 10 µL in one crystallization drop). Second, it was difficult to generate reproducible homogenous and singular microcrystals. Lastly, the diffusion method took several hours to obtain the microcrystals; thus, it was not easy to use excessive crystallization drops at once. Therefore, a batch method in a microtube^37–40^ was applied to overcome these difficulties. We increased the protein concentration to approximately ten times that of the previous trials and optimized the crystallization conditions with various trials. Finally, we reproduced millions of microcrystals by simple mixing the proteins and the crystallization solution in the microtube (Fig. 1a).

After mixing the proteins with the precipitants, we observed the crystalline slurry immediately. An hour was sufficient for obtaining the crystals with approximate dimensions of 20 × 20 × 50 *μ*m^3^ (Fig. 1b). The micron-size crystals were easily reproduced homogenously in a nucleated single form without cracks or disruptions. The hemocytometer indicated that the number of microcrystals was 1–2 × 10^6^ crystals/ml (Fig. 1b). We further checked the homogeneity and population of cellular-size crystals using flow cytometry (Supplementary Fig. S1). The microcrystals exhibited a monodisperse peak and a total of 2.2 × 10^6^ crystals/ml in the histogram of forward scattering *vs*. count. This indicated that the current microcrystals were suitable for SFX experiments.

### Structure determination using XFEL

We mixed microcrystals in a viscous medium as a carrier matrix and injected using LCP injector. The microcrystals were stable and well diffracted in a monoolein mixture. Then, we successfully collected a total of 232,694 images at RT. The average hit rate was approximately 12.5%, and the number of hit images was 28,966. The diffraction images were indexed by optimizing the detector geometry. Finally, we indexed a total of 22,925 images. The indexing rate was as high as 79.1%. The space group was *I222*, and the distribution of the unit cell parameters was a = 55.86 Å, b = 74.97 Å, and c = 157.62 Å. Compared with the cell parameters in a cryogenic condition^34^, the c-axis was extended to approximately 3 Å.

**Figure 2:**
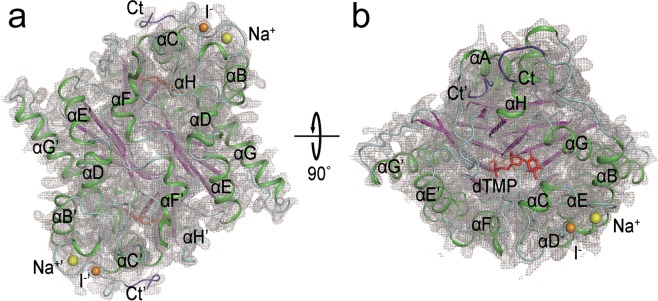
XFEL structure of dimeric T4dCH D179N within electron density map (gray) projected along twofold rotational axis of symmetry. (**a**) Front and (**b**) top view rotated along x-axis by 90°. 2Fo-Fc maps (gray) are contoured at 1.0 σ. Secondary structural elements are drawn as ribbon diagram in green (α- and 3_10_-helix), purple (β-strand), and cyan (loops). C-terminal ends are colored blue and labeled as Ct (N-terminal ends exist in behind side). Bound ligands are shown using ball-and-stick model in red (dTMP), orange (iodide), and yellow (sodium ion).

The number of unique reflections was 27,244, and the redundancy was as high as 595.9. The overall R_split_ was 19.14%, and the overall signal-to-noise ratio (SNR) was 4.87. The T4dCH D179N microcrystals were diffracted up to approximately 1.9 Å, and the SNR of the highest-resolution shell (1.95–1.88 Å) was 2.51 near the edge of the detector. At the highest-resolution shell, the Pearson correlation coefficient (CC) and CC* values were 0.80 and 0.94, respectively. After processing the entire dataset with a completeness of 100%, we solved the XFEL structure of T4dCH D179N at a resolution of 1.9 Å (Fig. 2). At the end of the refinement, the model yielded a R_work_/R_free_ value of 0.169/0.191. Details of the data collection and refinement are listed in Table 1.

**Table 1:** Data collection and refinement statistics.

	D179N mutant
**Data collection**	
X-ray source	PAL-XFEL (SFX)
Energy (eV)	9,700
Pulse width (fs)	20
Space group	*I*222
Cell dimensions	
*a*, *b*, *c* (Å)	52.86, 74.97, 157.62
α, β, γ (°)	90, 90, 90
No. of total collected images	232,694
No. of diffracted images	28,963
No. of indexed images	22,925
No. of total reflections	16,234,836
No. of unique reflections	27,244
Resolution (Å)	40.0–1.88 (1.95–1.88)^a^
*R*_split_ (%)^b^	19.14 (37.95)
<*I/σ* (*I*)>	4.87 (2.51)
CC^c^	0.93 (0.80)
CC*^d^	0.98 (0.94)
Completeness (%)	100.0 (100.0)
Redundancy	595.9 (176.2)
**Refinement**	
Resolution (Å)	37.50–1.90 (1.97–1.90)
No. reflections	26,553
*R*_work_/*R*_free_ (%)	16.91/19.05 (21.08/22.90)
No. atoms	
Protein	2,009
Ligand (dTMP, I, Na) / Water	23 (21, 1, 1) / 60
B-factors (Å^2^)	
Protein	58.4
Ligand (dTMP, I, Na)/Water	50.6 (50.6, 50.4, 50.4) / 54.9
RMSDs	
Bond lengths (Å)	1.286
Bond angles (°)	0.019
Ramachandran plot (%)	
Favored	96.72
Allowed	3.28
Outliers	0
Clashscore	1.14
MolProbity score	1.75

^a^Values in parentheses are for highest-resolution shell.

^b^*R*_split_ = 2^−1/2^ × ∑|I_even_ − I_odd_|/[1/2 × ∑ (I_even_ + I_odd_)].

^c^Pearson correlation coefficient.

^d^CC* = [2CC_1/2_/(1 + CC_1/2_)]^1/2^.

### Overall structure of T4dCH D179N mutant

**Figure 3:**
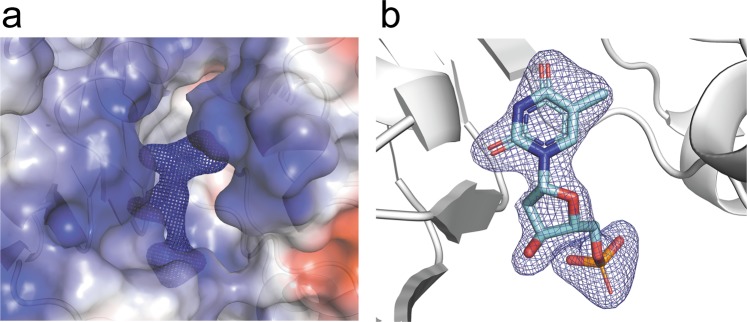
Electron density map of initial refinements in substrate binding site of T4dCH D179N XFEL structure. Fo-Fc map is contoured at 3.0 σ. (**a**) Electron density map with electrostatic surface model and ribbon diagram of T4dCH D179N. (**b**) Fitting dTMP molecule into electron density map.

The overall model of T4dCH D179N fitted in well with an electron density map generated by XFEL (Fig. 2). The polypeptide chains were clearly visible from the N-terminus of Met1 to the C-terminus of Ala246, except for the additional C-terminal histidine tag (Supplementary Fig. S2a,b). Near the active site, a flexible loop around Lys28 residue for covering the bound substrate fitted in well with the electron densities (Supplementary Fig. S2c). We could also observe the clear electron density for the side chains of catalytic key residues (Supplementary Fig. S2d). Intriguingly, a bulky difference map was detected in the substrate binding site of the XFEL structure even though no substrate-like compounds were added during the purification and crystallization steps (Fig. 3). We assumed that this molecule may be dTMP. This is further detailed in the following section.

We found two blubs around 3_10_-helix G1 that were interpreted as an iodide and a sodium ion (Supplementary Fig. S3a). As mentioned in our previous report^34^, the iodide ion affected the crystal contacts of space group *I222*, which was clearly implied by the Fo-Fc difference map. The binding of a sodium ion was not discussed in previous studies because the software automatically predicted this position as a water molecule. We carefully explored the XFEL structure and found that this electron density matches with the sodium ion that forms an octahedral geometry with the main chain oxygen atoms of Lys72, Ile74, and Gly76, and side chain hydroxyl group of Thr78, in addition to two water molecules (Supplementary Fig. S3b). The distances between the sodium ion and the oxygen atoms of protein and water molecules were well matched with the observed distance range as reported in the PDB data bank^41,42^.

### Unique features of RT structure

Next, we compared the XFEL structure of T4dCH D179N with our previous structures of *C2-*form crystals collected at RT (PDB ID: 1B5E) and *I222*-form crystals under cryogenic conditions (PDB ID: 6A9A). The calculated root-mean-square deviation (RMSD) value was 0.447 Å for 240 matching Cα atoms between the XFEL- and *C2-*form structures. Interestingly, despite having the same space group and similar cell parameters, the Cα RMSD between the XFEL- and *I222-* form structures in cryogenic conditions was 0.780 Å, which indicates more deviations than the previous one. The major difference between previous *C*2- and *I*222-form crystal structures was the presence or absence of hydrogen-bonding interactions of the N-terminus to the other protomer in the XFEL structure^33,34^.

The conformation of the N-terminal region of the XFEL structure was similar to that of the *C2* structure collected at RT. The hydrogen-bonding interactions were maintained by Met1-Asp23′, Met1-Glu35′, Met1-Ile37′, and Ser3-Ile37′ (apostrophe indicates another subunit of the dimer) (Supplementary Fig. S4). Therefore, there might be minor structural rearrangements in the N-terminal region when the hydrogen-bonding interaction is hindered during the freezing process. This is an excellent example of determining the protein structure at a more natural RT using XFEL technology.

### Molecule derived from host *E. coli* found at substrate binding site

The unknown electron density was occupied at the substrate binding site of T4dCH D179N (Fig. 3a). In the protein preparation and crystallization conditions, no ligand-like molecules were included. For the molecular replacement (MR) calculation, the template model did not include any ligand-like molecules. Therefore, the omit map seemed to represent the molecule that originated from *E. coli*, which is the physiological host of the T4 bacteriophage. Because the resolution of our XFEL structure was sufficiently high, we attempted to interpret a ligand molecule based on electron density. First, a bulky electron density was found between the guanidinium groups of Arg123′ and Arg124′ residue^33^. This is known as a binding site of a phosphate moiety (Supplementary Fig. S5a). Second, an electron density similar to the previous one^33^ existed between the ε-nitrogen of His216 and the hydroxyl group of Tyr218. This is known as the recognition site of a deoxyribose ring (Supplementary Fig. S5b)^33,43^.Lastly, a disc-shaped electron density filled the active cavity near the catalytic cysteine (Cys148) and the pyrimidine-determining residue (D179N) (Supplementary Fig. S5c). Moreover, it was very similar to the electron density of dCMP in the active site of the binary complex (PDB ID: 1B5E), except for the residual density at the C5 position of the pyrimidine base (Fig. 3b and Supplementary Fig. S5d). Thus, we assumed that the unknown ligand was a methylated pyrimidine nucleotide analog.

### Mass spectrometry analysis for ligand identification

**Figure 4:**
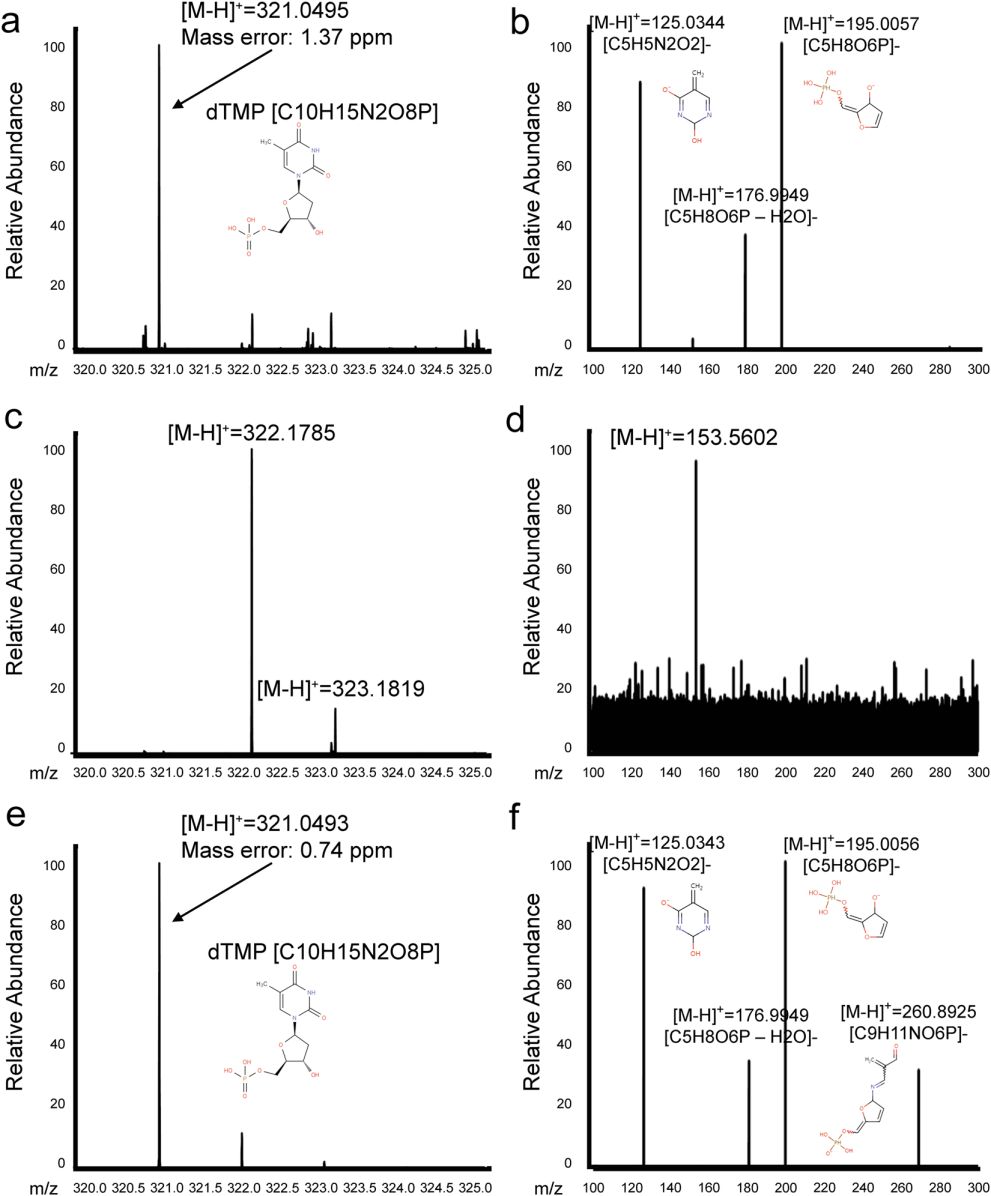
Identification of dTMP in T4dCH D179N and WT by high-resolution, high-mass-accuracy mass spectrometry. (**a**) Mass spectrum of T4dCH D179N. (**b**) Tandem mass spectrum of [M-H]^+^ = 321.0495 from T4dCH D179N. (**c**) Mass spectrum of T4dCH WT. (**d**) Tandem mass spectrum of [M-H]^+^ = 321.0495 from T4dCH WT. (**e**) Mass spectrum of standard dTMP. (**f**) Tandem mass spectrum of [M-H]^+^ = 321.0495 from standard dTMP.

For the accurate identification of the unknown nucleotide analog by qualitative analysis, we employed mass spectrometry for analyzing the analog (Fig. 4). Because the WT protein possessed an empty active site, we compared the mass spectral signals for T4dCH D179N and WT protein. The major difference in the signals between D179N and WT protein was found at 321.0495 m/z (Fig. 4a,c). Interestingly, the mass spectral signal of dTMP occurred at 321.0493 m/z and exactly matched the m/z ratio of the unknown substance in T4dCH D179N protein (Fig. 4a,e). For further confirmation, high-resolution collisional dissociation (HCD) fragmentation was analyzed. A fragment spectrum of m/z ratio 321.0495 in D179N was detected at the same positions of the dTMP fragments (Fig. 4b,f). Therefore, by combining XFEL crystallography and mass spectrometric analysis, the unknown substance bound to T4dCH D179N was revealed as a dTMP molecule.

### Native dTMP-bound structure of T4dCH D179N

**Figure 5:**
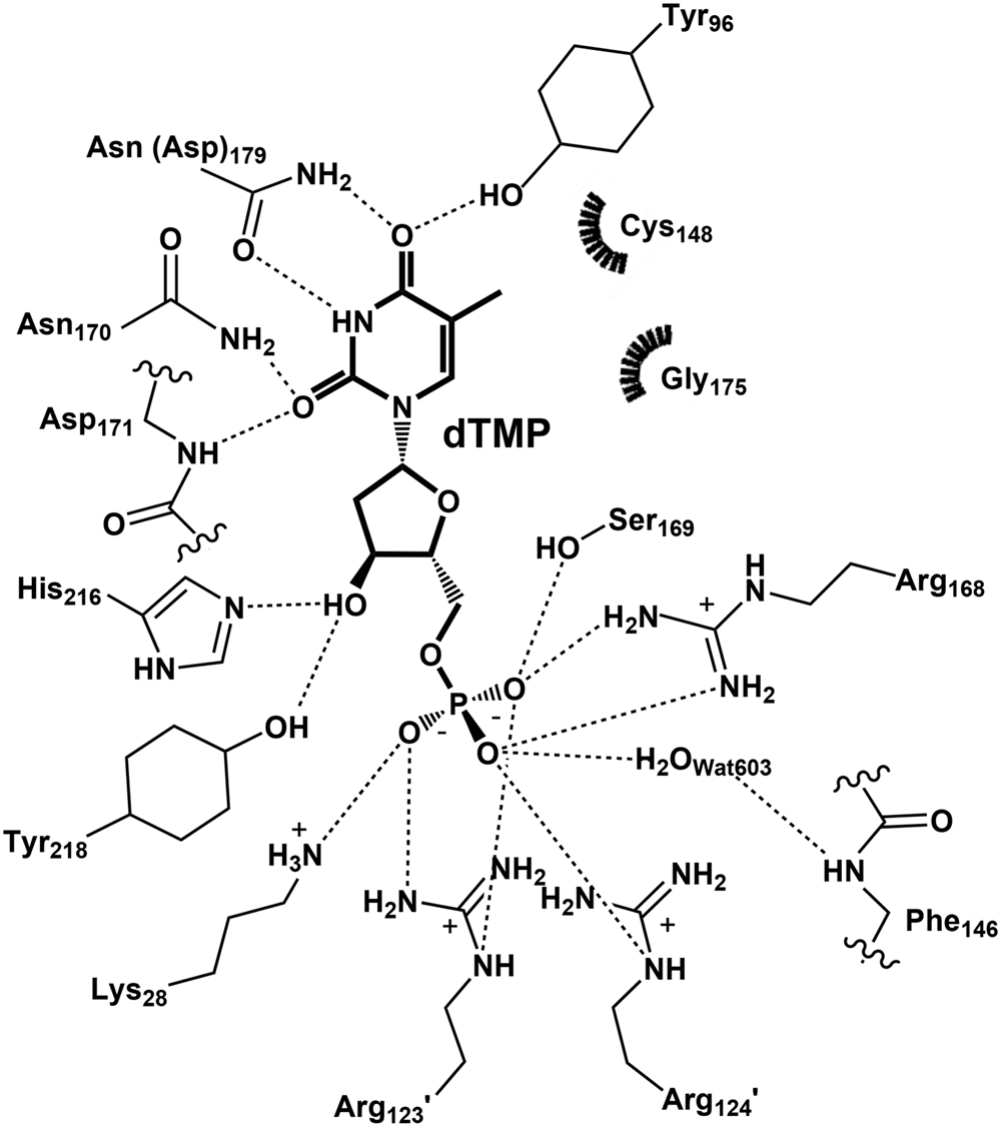
Schematic diagram of dTMP interaction. Hydrogen bonds are indicated by dashed lines, while hydrophobic contacts are represented by arc with spokes. Diagram was generated by LIGPLOT^55^ and modified for clarity.

The T4dCH D179N structure was further refined after incorporating the dTMP model at the active site. The dTMP molecule was well fitted to the electron density map in the active site (Fig. 3b). The overall ligand geometry of dTMP was similar to that of dCMP in the binary complex of T4dCH (Fig. 3b and Supplementary Fig. S5d). The details of the interaction between dTMP and T4dCH D179N are as follows. The phosphate moiety of dTMP was recognized by the side chain atoms of Lys28, Arg168, Ser169, Arg123′, Arg124′, and a water molecule (Wat603), which formed a hydrogen bond with the main-chain atom of Phe146 (Fig. 5). A hydroxyl group of deoxyribose moiety was recognized by the side chain atoms of His216 and Tyr218.

For the recognition of the thymine base, the carbonyl oxygen at position 2 was recognized by the side-chain nitrogen atom of Asn170 and the main-chain nitrogen atom of Asp171, and the carbonyl oxygen at position 4 was recognized by the side-chain oxygen atom of Tyr96 and side-chain nitrogen atom of Asn179. Interestingly, the Asn residue of the mutant recognized the N3 atom and the carbonyl oxygen at position 4 in the thymine base, whereas the carboxylate of Asp179 of WT interacted with the N3 atom and primary amine group at position 4 of the cytosine base; this is shown in the binary complex structure^33^. In comparison with the ligand geometry of dCMP in the binary complex of T4dCH WT, the ligand geometry of dTMP in the XFEL structure of D179N exhibited a critical difference around a methyl group at the C5 position in the pyrimidine base. Indeed, the C5 position is where the hydroxymethylation occurs, and the previous study suggested that there is a methylene intermediate at this position^34^. Therefore, our dTMP-bound structure provided an insight into the methylene intermediate structure.

### Mimicry of methylene intermediate state

Our previous studies on the hydroxymethylation mechanism of T4dCH suggested that the hydride transfer found in the enzymatic reaction of TS was blocked because the distance from the C6 of THF to the putative C7 exocyclic methylene position^34^ was too large. Instead, the reaction of T4dCH underwent hydroxylation by the potential substrate water molecule near the putative C7 exocyclic methylene position^34^. This C7 methyl group must be transferred, and it must form a covalent bond with the C5 atom of the cytosine base. Our dTMP-bound structure well mimicked the methylene intermediate that was the methylated pyrimidine nucleotide complex (Supplementary Fig. 6). Therefore, we tried to understand the final hydroxylation reaction compared to the dTMP-bound binary complex with ternary complexes of T4dCH and EcTS (PDB IDs: 6A9A and 1KZI)^34,44^.

**Figure 6:**
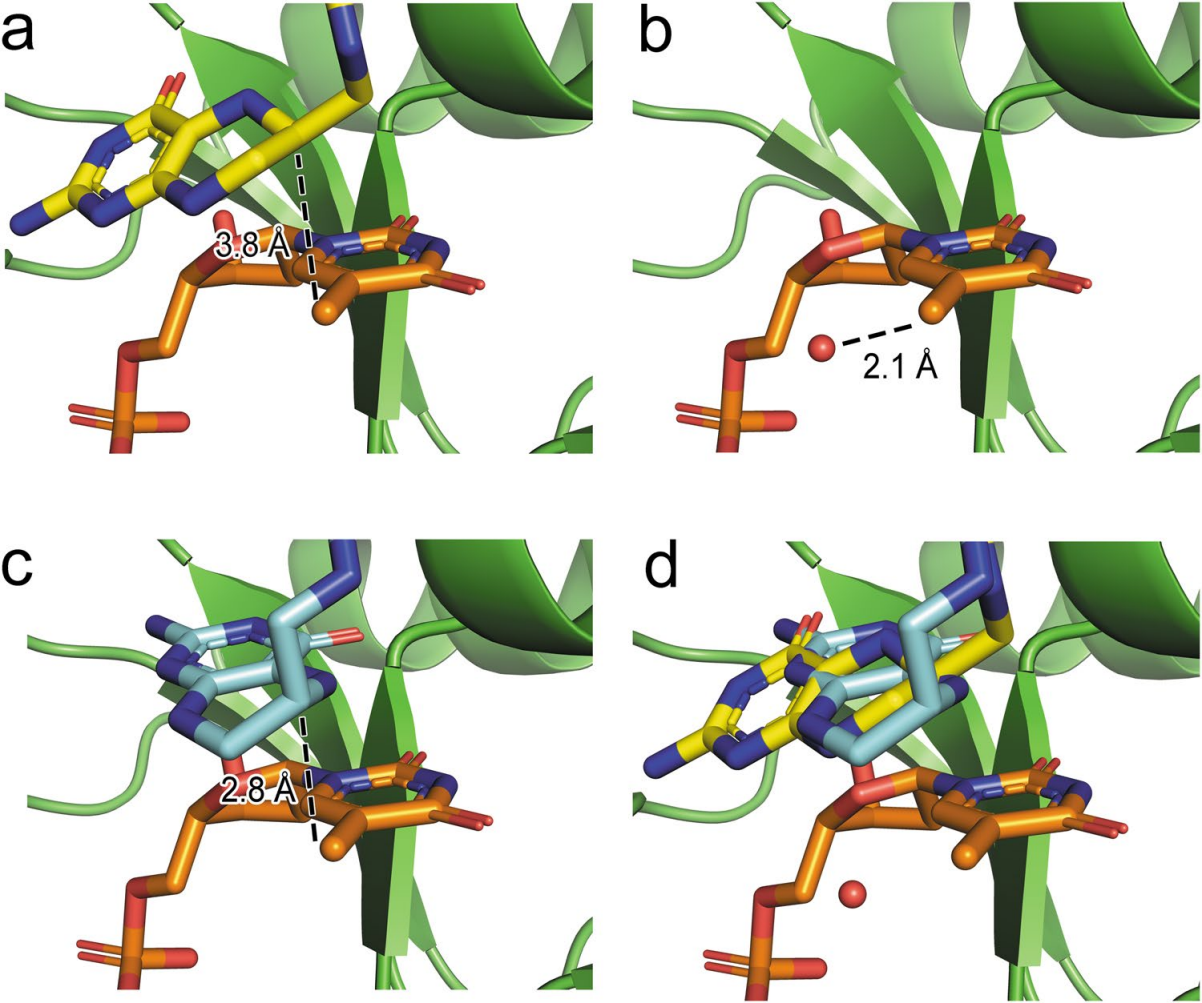
Mimicry of methylene intermediate by dTMP-bound T4dCH D179N mutant. Superposition between current T4dCH mutant (green) complexed with dTMP (orange) and (**a**) THF (yellow) in ternary complex of T4dCH, (**b**) potential substrate water (red) in ternary complex of T4dCH (Wat401 in PDB 6A9A), and (**c**) THF in ternary complex of EcTS (cyan). (**d**) Superimposed models of panels (a–c). Models are shown by ribbon diagram and stick model. In stick model, atoms are colored red (oxygen) and blue (nitrogen).

When the dTMP-bound complex was superimposed on the ternary [T4dCH·dCMP·THF] complex, the C7 position of dTMP and the C6 position of THF were at a distance of 3.8 Å from each other (Fig. 6a), whereas the C7 position of dTMP was at a distance of 2.1 Å from a potential substrate water molecule (Wat401 in 6A9A) (Fig. 6b). Indeed, the current structure exhibited an electron density for a water molecule (Wat601) that may correspond to the Wat401 in the ternary complex structure^34^ (see next section for more details). In contrast, by superimposing the dTMP-bound complex and the ternary complex [EcTS·dUMP·THF], the C7 position of dTMP and C6 position of THF were within a distance of 2.8 Å, which is much less than 3.8 Å (Fig. 6c). The hydrogen atom attached to the C6 position of THF transferred to the methylene intermediate. This was the rate-limiting hydride transfer step of the TS enzymatic mechanism^44,45^. The main difference was derived from the different orientation of the THF cofactor (Fig. 6d).

### Water molecules at dTMP-bound region

Six water molecules (Wat601–606) were built with reliable electron densities at the active site of the intermediate-mimicking structure (Supplementary Fig. 7). All water molecules were maintained by hydrogen-bonding interactions with the amino acid residues of the active site pocket. Compared with the binary and ternary complex structures (PDB IDs: 1B5E and 6A9A), most of the water molecules were well ordered (Wat602–606); however, a water molecule was newly observed (Wat601) near the Asp145 residue. The position of this water molecule did not overlap that of the previously proposed substrate water molecule (Wat401 in 6A9A).

However, both Wat601 in the current structure and Wat401 in the ternary complex were equidistant (2.8 Å) from the Asp145 residue. The electron density of the new water molecule was relatively weak than those of other solvents because it was stabilized by hydrogen-bonding interactions with the carboxylate of Asp145 only. Despite a weak interaction, Wat601 existed at a very close distance from the previous substrate water molecule in the ternary complex structure (within 2.0 Å). Therefore, the substrate water molecule existed near the carboxyl group of Asp145 residue at the methylene intermediate stage. Then, the molecule was activated and moved to a position closer to the C7 of the exocyclic methylene intermediate by hydrogen-bonding interactions of Asp145 and THF. As noted earlier, all other water molecules were structurally conserved except for Wat601. The previous study proposed that another water molecule (Wat409 in 6A9A) plays an important role in the activation of the dCMP substrate^34^. This step occurs before the formation of the methylene intermediate. The position of Wat602 in the current structure overlapped that of Wat409 in the ternary complex. Thus, a water molecule (Wat602) for substrate activation existed constitutively at the fixed position, whereas a water molecule (Wat601) involved in the final hydroxylation step was located at a slightly different position that could be adjusted during the enzymatic reaction steps of T4dCH.

In conclusion, the current dTMP-bound structure provides clear experimental support for the previously proposed hydroxylation step of T4dCH^34^. Owing to the space constraint of the ternary [EcTS·dUMP·THF] complex, no water molecule could be accommodated near the cofactor and substrate, whereas a water molecule found in the ternary complex [T4dCH·dCMP·THF] was located at the optimal position near the methylene intermediate for hydroxylation. Compared to the hydride transfer of TS, the last hydroxylation step of dCH could be directly monitored by the movement of the electron density of oxygen atoms including the substrate water molecule in a crystal structure. Further TR-XFEL study using a rapid mix-and-inject technique will allow us to visualize the molecular motion of the hydroxymethylation mechanism.

## Materials and Methods

### Expression and protein purification

T4dCH D179N mutant was generated by PCR-based mutagenesis, which has been described in a previous paper in detail^34^. The protein was expressed in *E. coli* BL21(DE3) cells with a C-terminal octa-histidine affinity tag to maximize the protein yield. The cells were grown overnight in LB media and then induced with 0.5 mM of isopropyl-β-D-1-thiogalactopyranoside (IPTG) for 4 h at 37 °C. Subsequently, the cells were harvested and resuspended in buffer A [50 mM Tris-HCl pH 8.0, 150 mM NaCl, and 1 mM tris(2-carboxyethyl)phosphine)] with 10 mM of imidazole. The resuspended cells were disrupted by sonication and then centrifuged (17,000 rpm for 1 h at 4 °C) to remove the cell debris.

Supernatants were loaded onto a Histrap FF (GE Healthcare, 17-5255-01) and eluted with gradually increasing concentrations of imidazole up to 500 mM in buffer A. The eluents were concentrated using an Amicon Ultra-30K centrifugal filter unit (Millipore, UFC903024) and dialyzed against a dialysis buffer (13.6 mM Na_2_HPO_4_, 8.1 mM NaH_2_PO_4_, 1 mM ethylenediaminetetraacetic acid, 150 mM NaCl, and 1 mM 1,4-dithiothreitol) using a Slide-A-lyzer MINI Dialysis Device (Thermo Fisher Scientific, 88405) at 4 °C overnight. The final concentration of purified protein was 40 mg/ml.

### Microcrystal preparation

Microcrystals of T4dCH D179N mutant were grown by the free-interface diffusion (FID) method in a 1.5-ml microtube (Axygen, MCT-150-C). The crystallization condition was initially attempted from the wild-type crystal condition reported previously^33^. The optimized crystallization solution [2 μl of 1.0-M Tris-HCl (pH 8.5), 2 μl of 1.0-M NaI (Sigma, 383112), and 17 μl of C_6_H_5_Na_3_·2H_2_O (Hampton Research, HR2-549)] and 10 μl of 40 mg/ml D179N mutant protein were mixed gently to avoid air bubbles. Microcrystals of approximately 20 × 20 × 50 *μ*m^3^ were grown at 20 °C within 1 h. The homogeneity and morphology were determined by a hemocytometer (Marienfeld-Superior, 0610030 and INCYTO, DHC-N01) and a flow cytometer (BD, BD Accuri C6 Plus). For SFX experiments, 40 μl of microcrystal solution was mixed with 60 μl of monoolein (Hampton Research, HR2-435) using syringes (Hamilton, 81065-1710RNR and 81165-1725RNR) and an LCP coupler (Mitegen, SKU:M-R-1006905). The crystal population of the monoolein mixture was monitored using a light microscope.

### Data collection and processing

The diffraction data were collected at a Nanocrystallography and Coherence Imaging (NCI) hutch at PAL-XFEL using an LCP injection system^46^. The X-ray energy was 9.7 keV with a repetition rate of 30 Hz and duration of 20 fs. The pulse energy was approximately 485 μJ, and the photon flux was approximately 1–2 × 10^11^ photons per pulse. The incident X-ray beam was focused at 5 × 5 μm^2^ full width at half maximum (FWHM) by Kirkpatrick-Baez mirrors^47^. The sample was delivered to the X-ray pulse in a helium ambience at RT in a multifarious injection chamber of a molecular structure study (MICOSS) system^48^. The diffraction data were obtained on an MX225-HS (Rayonix LLC) CCD detector with 4 × 4 binning mode corresponding to a pixel size of 156 μm. The detector was positioned 108 mm from the sample. The edge of the detector had a resolution of approximately 1.8 Å. The serial X-ray images were monitored in real time using OnDA^49^, and the diffraction images were sorted using the Cheetah software with the ‘peakfinder 8’ algorithm^50^. The filtered images were indexed using *indexamajig* in the CrystFEL package^51^. The indexed images were scaled and merged by *partialator*. The data quality was calculated by *check_hkl* and *compare_hkl* in the CrystFEL package. The total number of collected images/hits/indexed images was 232,694/28,963/22,925 (Table 1).

### Structure determination, ligand fitting, and refinement

The phases were obtained by molecular replacement (MR) using *Phaser*^52^ with the previously solved wild-type structure as a search model (PDB ID: 1B5E)^33^. To model the dTMP as a ligand, the eLBOW module in PHENIX was used with ligand geometric constraints. Using the LigandFit module in PHENIX, the 21 atoms of dTMP without hydrogens were well fitted into the difference map with an overall CC and score of 0.852 and 73.10, respectively. Refinement and validation of the dTMP-bound model were performed using COOT^53^ and PHENIX^54^. For interface analysis of the ligand-protein contacts, LIGPLOT^55^ and the PISA server in the CCP4 suite^56^ were used. All structural figures were prepared using PyMOL (http://www.pymol.org).

### Mass spectrometry

Mass spectrometry was performed with a Q Exactive Plus mass spectrometer (Thermo Fisher Scientific, IQLAAEGAAPFALGMAZR). Samples dissolved in 0.1% formic acid were infused with a syringe at a flow rate of 5 µl/min. A mass spectrometer equipped with a microspray ESI source was operated in full mass-spectral-acquisition mode for MS scan data in a mass range of m/z 100–500. A resolving power setting of 70,000 was used in negative ion mode. MS/MS scan data were also acquired in negative ion scan mode; a normalized collision energy (NCE) of 35% was applied to a precursor ion selected, m/z 321.05. The MS and MS/MS scan AGC target value was set at 1e^6^. All data acquisition and analysis were carried out with XCalibur v.2.2 software (Thermo Fisher Scientific).

### Accession code

Atomic coordinates and structure factor files for T4dCH D179N have been deposited in the Protein Data Bank under the accession code 6L18.

## Supplementary information

Supplementary information
