# Supplementary material for: A host dTMP-bound structure of T4 phage dCMP hydroxymethylase mutant using an X-ray free electron laser
Source: Sci Rep. 2019 Nov 8;9:16316. doi: 10.1038/s41598-019-52825-y (PMC6841964; doi:10.1038/s41598-019-52825-y)
Supplement: Supplementary file 1 — Supplementary information [file 41598_2019_52825_MOESM1_ESM.pdf]

Supplementary Figures

A host dTMP-bound structure of T4 phage dCMP hydroxymethylase mutant using an X-ray free electron laser

Si Hoon Park[1], Jaehyun Park[2], Sang Jae Lee[2], Woo Seok Yang[1], Sehan Park[2], Kyungdo Kim[3], Zee-Yong Park[3], and Hyun Kyu Song[1,*]

[1]*Department of Life Sciences, Korea University, 145 Anam-ro, Seongbuk-gu, Seoul 02841, South Korea*

[2]*PAL-XFEL, Pohang Accelerator Laboratory, POSTECH, Pohang, Gyeongbuk 37673, South Korea*

[3]*School of Life Sciences, Gwangju Institute of Science and Technology, Gwangju 61005, South Korea*

[a] Correspondence: Hyun Kyu Song, Department of Life Sciences, Korea University, 145 Anam-ro, Seongbuk-gu, Seoul 02841, South Korea, Tel: 82-2-3290-3457, Fax: 82-2-3290-3628, E-mail: hksong@korea.ac.kr

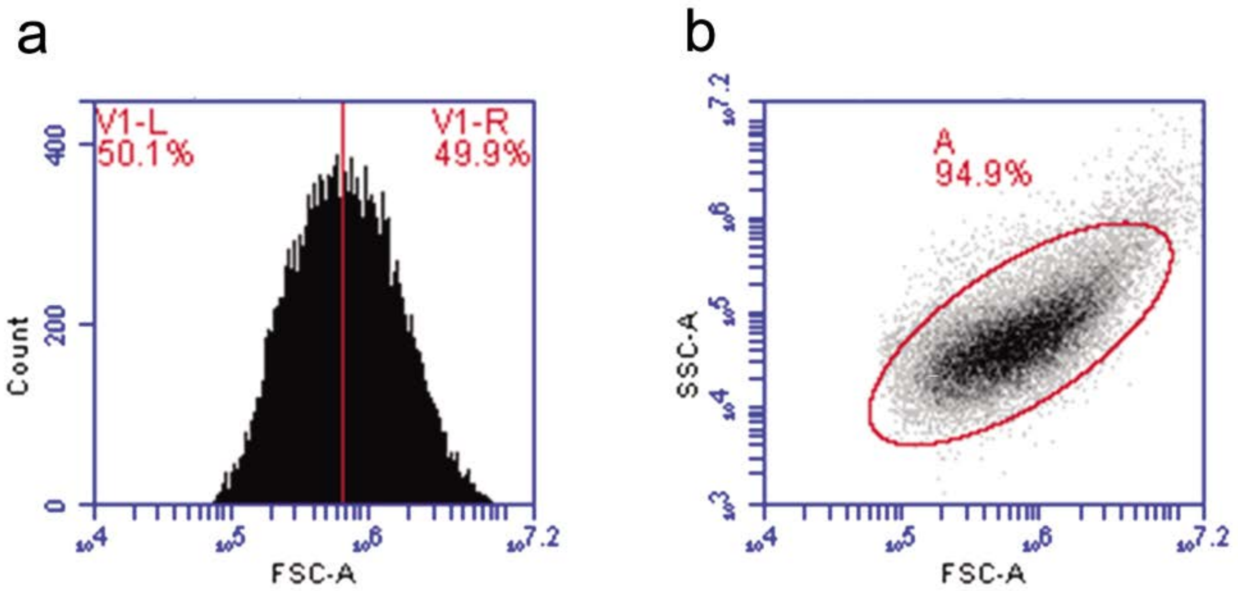

Supplementary Figure S1. Flow cytometry of T4dCH D179N microcrystals. (a) Histogram of microcrystals in forward scatter (FSC) *vs.* count. (b) Dot plot of microcrystals in forward scatter (FSC) *vs.* side scatter (SSC). All axes are shown on logarithmic scale except count axis in panel (a).

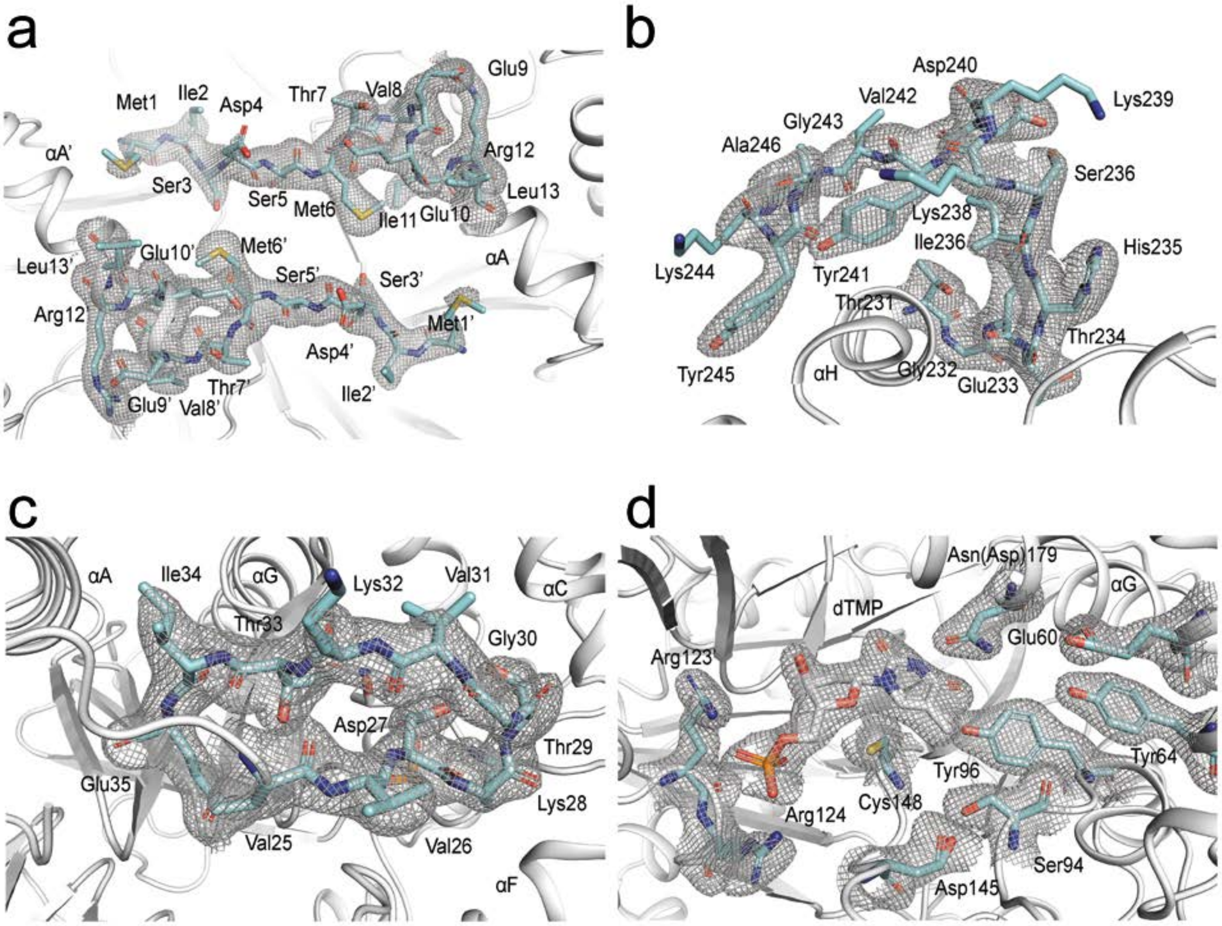

Supplementary Figure S2. Quality of electron density (gray) produced by XFEL experiments. Electron density map around (a) N-terminal Met1 residue, (b) C-terminal Ala246 residue, (c) loop near Lys28 residue, and (d) catalytic key residues. 2Fo-Fc map is contoured at 1.0 σ. Overall structures are shown by ribbon diagram (white) and stick models colored with cyan (carbon), red (oxygen), blue (nitrogen), and yellow (sulfur).

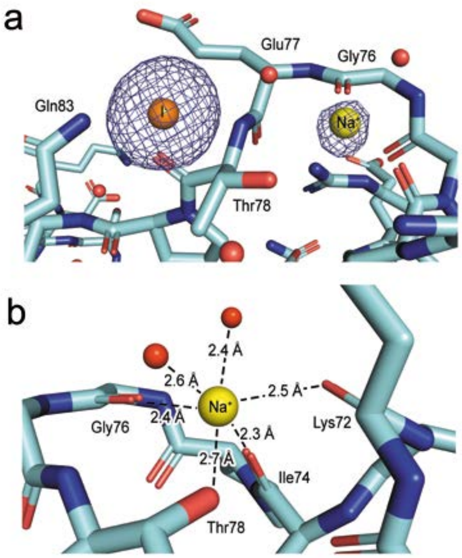

Supplementary Figure S3. Location of iodide and sodium ion in XFEL structure of T4dCH D179 N. (a) Electron density maps of iodide and sodium ion. (b) Coordination of sodium ion is an octahedral geometry. Iodide ion (orange), sodium ion (yellow), and water molecules (red) are shown as sphere model. Fo-Fc map (blue mesh) is contoured at 5.0 σ. Polypeptide chains are shown as stick model colored with cyan (carbon), red (oxygen), and blue (nitrogen). Dashed lines with distance indicate interactions between sodium ion and ligand oxygen atoms.

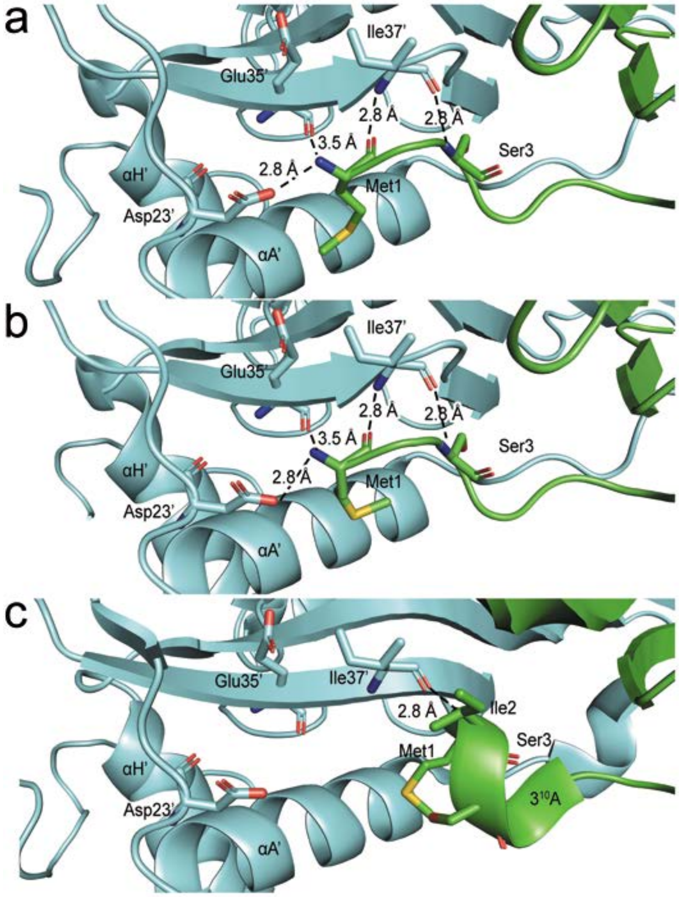

Supplementary Figure S4. Hydrogen bonding interaction between N-terminus of T4dCH (green) and other protomer (cyan). (a) XFEL structure of T4dCH D179N. (b) *C2* form crystal structure of WT (PDB ID: 1B5E). (c) *I222* form crystal structure of C148S/D179N (PDB ID: 6A9A). Models are shown by ribbon diagram and stick model colored with red (oxygen), blue (nitrogen), and yellow (sulfur).

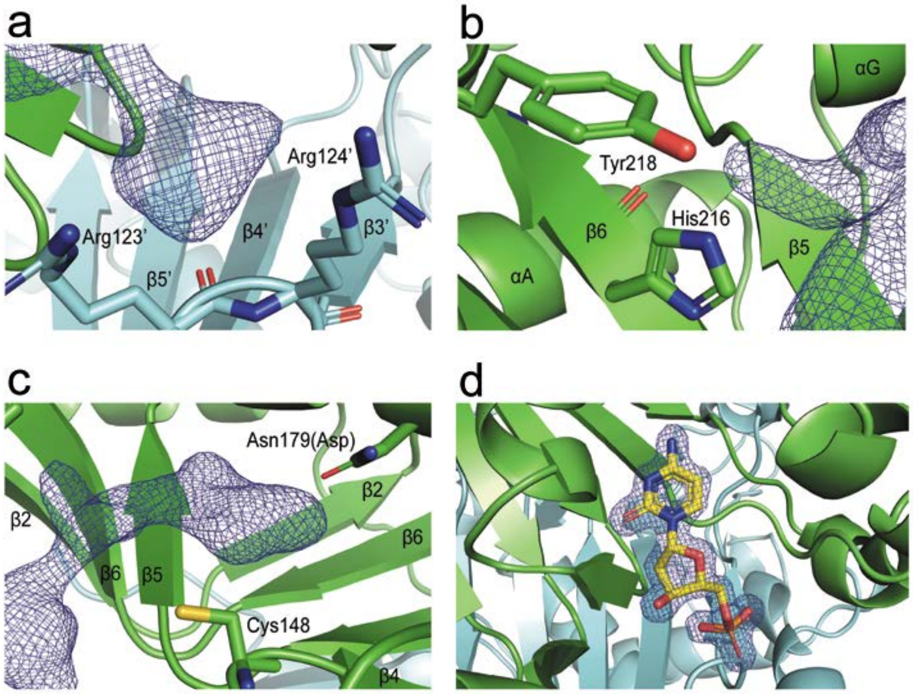

Supplementary Figure S5. Key residues near electron density of unknown ligand in active site of T4dCH D179N. Electron density map (a) around Arg123' and Arg124' residue, (b) around His216 and Tyr218 residue, and (c) around Asn179(Asp) and Cys148 residue. (d) Electron density map of dCMP in binary complex of T4dCH (PDB ID: 1B5E). Models are shown by ribbon diagram and stick model. Each chain of dimer is colored green and cyan, respectively. In stick model, atoms are colored red (oxygen), blue (nitrogen), and yellow (sulfur).

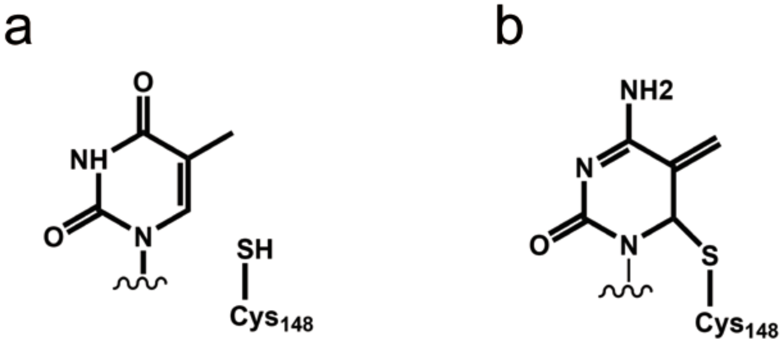

Supplementary Figure S6. Mimicry of methylene intermediate. (a) Thymine base of dTMP-bound structure of T4dCH N179D mutant. (b) Methylene intermediate of dCMP based on reaction mechanism.

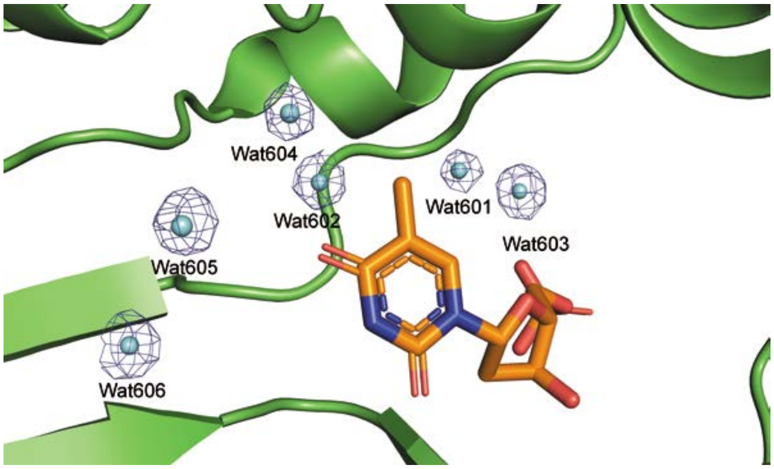

Supplementary Figure S7. Water molecules in active site of T4dCH mutant. 2Fo-Fc electron density map (blue) is contoured at 1.0 σ. Models are shown by ribbon diagram of T4dCH D179N (green) and stick model of dTMP (orange). Water molecules are colored cyan, and nitrogen and oxygen atoms in dTMP are colored blue and red, respectively.
